# The FOXO1 inhibitor AS1842856 triggers apoptosis in glioblastoma multiforme and basal‐like breast cancer cells

**DOI:** 10.1002/2211-5463.13547

**Published:** 2023-01-16

**Authors:** David Flores, Alma Lopez, Shreya Udawant, Bonnie Gunn, Megan Keniry

**Affiliations:** ^1^ Department of Biology University of Texas‐Rio Grande Valley Edinburg TX USA

**Keywords:** apoptosis, AS1708727, AS1842856, basal‐like breast cancer, glioblastoma multiforme

## Abstract

Basal‐like breast cancer (BBC) and glioblastoma multiforme (GBM) are poor‐prognosis cancers that lack effective targeted therapies and harbor embryonic stem gene expression signatures. Recently, our group and others found that forkhead box transcription factor FOXO1 promotes stem gene expression in BBC and GBM cell lines. Given the critical role of cancer stem cells in promoting cancer progression, we examined the impact of FOXO1 inhibition with AS1842856 (a cell‐permeable small molecule that directly binds to unphosphorylated FOXO1 protein to block transcriptional regulation) on BBC and GBM cell viability. We treated a set of BBC and GBM cancer cell lines with increasing concentrations of AS1842856 and found reduced colony formation. Treatment of BBC and GBM cancer cells with AS1842856 led to increases in *FAS* (*FAS cell surface death receptor*) and *BIM* (*BCL2L11*) gene expression, as well as increased positivity for markers for apoptosis such as annexin V and propidium iodide. Treatment with another FOXO1 inhibitor AS1708727 or *FOXO1* RNAi also led to *FAS* induction. This work is the first to show that targeting BBC and GBM with FOXO1 inhibition leads to apoptosis. These novel findings may ultimately expand the repertoire of therapies for poor‐prognosis cancers.

## Graphical abstract

Glioblastoma multiforme (GBM) and basal‐like breast cancer (BBC) harbor FOXO1‐driven embryonic stem gene expression signatures. Treatment of GBM and BBC cells with the FOXO1 inhibitor AS1842856 led to increased pro‐apoptotic gene expression, increased apoptosis, and reduced colony formation. This work is the first to show that inhibiting FOXO1 in BBC and GBM leads to apoptosis, highlighting a novel targeted therapy.

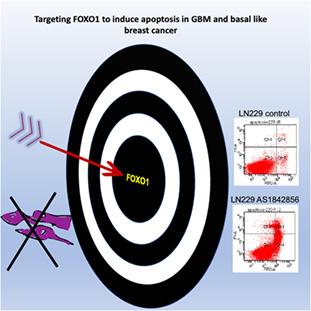

**Abbreviations**
*5‐AZA*
: *5*‐*aza*‐2′‐deoxycytidine
AML: acute myeloid leukemia
AML: acute myeloid leukemia
BBC: basal‐like breast cancer
BIM: BCL2L11
DLBCL: diffuse Large B‐cell lymphoma
DMEM: Dulbecco's Modified Eagle Medium
EGFR: epidermal growth factor receptor
FAS: Fas cell surface death receptor
FOXO1: forkhead box transcription factor 1
GBM: glioblastoma multiforme
MEM: minimal essential medium
OCT4: POU class 5 homeobox 1
P27: CDKN1B
PI3K: phosphatidylinositol 3 kinase
PIP2: phosphatidylinositol 4,5 bisphosphate
PIP3: phosphatidylinositol 3,4,5 trisphosphate
PTEN: phosphatase and Tensin homolog located on chromosome ten
RPMI: Roswell Park Memorial Institute 1640 Medium
RTK: receptor tyrosine kinase
SOX2: SRY‐box transcription factor 2
TRAIL: TNFSF10
TUBB: tubulin beta class I

Basal‐like breast cancer (BBC) and glioblastoma multiforme (GBM) are aggressive cancers associated with poor prognosis [1, 2]. Weinberg and colleagues first discovered through gene expression profiling that BBC and GBM harbor embryonic stem‐like gene expression signatures [3]. In embryonic stem cells (ESCs), forkhead box transcription factor Foxo1 directly binds to stem genes to activate their transcription. Subsequent work by our group and others revealed that the forkhead box transcription factor FOXO1 also helped induce stem gene expression in examined BBC and GBM cell lines, highlighting conserved mechanisms in ESCs and cancer cells [4, 5]. FOXO1 induced the *OCT4* (*POU class 5 homeobox 1*) gene expression in glioblastoma cells [4]. Reduction of *FOXO1* and *FOXO3* transcription factors led to decreased protein expression of SOX2 (SRY‐box transcription factor 2), and NESTIN in patient‐derived GBM models [5]. Furthermore, FOXO transcription factors sustain stem cells in various contexts, including embryonic, hematopoietic, and neural [6, 7, 8]. The full spectrum of contributions that FOXO factors harbor in stem cell contexts remains to be fully delineated.

The Phosphatidylinositol 3 Kinase (PI3K) Pathway promotes cell growth, proliferation, and migration in BBC and GBM cells [9, 10]. Receptor tyrosine kinases (RTKs) such as epidermal growth factor receptor (EGFR) are bound by ligands, leading to dimerization and auto‐phosphorylation [11]. This creates docking sites on the RTKs that, among other things, activate the lipid kinase PI3K, which phosphorylates phosphatidylinositol 4,5 bisphosphate (PIP2) on the D3 position to produce phosphatidylinositol 3,4,5 trisphosphate (PIP3) [9, 10]. Lipid second messenger PIP3 binds to and activates targets such as AKT to promote growth and survival. AKT has over 20 identified targets, including FOXO1, ‐3, and ‐4 transcription factors on conserved residues, typically leading to their cytoplasmic sequestration/inactivation [12, 13]. However, a subset of FOXO transcription factors resides in the nucleus via unknown mechanisms in BBC and GBM despite constitutively active PI3K Pathway activity [14].

Epigenetics and mutations lead to nearly uniform constitutively active PI3K Pathway activity in BBC and GBM [15, 16, 17]. Commonly the dual‐specificity phosphatase *PTEN* (*Phosphatase and Tensin homolog located on chromosome ten*, which encodes a lipid phosphatase to diminish cellular pools of PIP3), is mutated to an inactive form in BBC and GBM [15, 16]. *EGFR* is frequently mutated to a constitutively active form in these cancers [18]. These changes significantly contribute to cancer formation, progression, and therapeutic resistance [16, 19].

Conserved FOXO1, ‐3, and ‐4 transcription factors are partially redundant and negatively regulated by AKT [20, 21]. These factors act in a context‐dependent manner to regulate metabolism by activating gluconeogenesis and impacting mitochondrial function [22, 23, 24]. The best‐described role for FOXO factors in cancer is to serve as tumor suppressors that induce genes such as *TRAIL* (*TNFSF10*) to promote apoptosis and *p27* (*CDKN1B*) to halt the cell cycle [22, 23]. Emerging evidence points to pro‐oncogenic roles for some FOXO factors in cancers such as diffuse large B‐cell lymphoma (DLBCL), in which *FOXO1* is commonly mutated to a constitutively nuclear form; these *FOXO1* mutations were associated with poor prognosis [25]. FOXO factors can promote breast cancer progression and therapeutic resistance [26, 27, 28]. These factors also impact stem programs in BBC and GBM, suggesting they might promote aggressiveness and could be putative therapeutic targets for these cancers [4, 5].

Basal‐like breast cancer has characteristics in common with myoepithelial cells of the breast and is typically triple negative: lack of the expression of the estrogen receptor, progesterone receptor, and HER2 receptor [29]. This breast cancer is commonly found in younger and African American women [29]. BBC is specifically associated with poor‐prognosis and chemotherapeutic resistance [29]. In terms of therapeutics, BBC is frequently triple negative and therefore responds only to conventional chemotherapy [30]. GBM is an aggressive brain cancer with a 5‐year survival rate of 6.8% [2]. GBM patients, on average have a survival length between 12 and 18 months [2]. Of all malignant brain tumors, GBM is the most common type found in adults. This study specifically focused on BBC and GBM, because both cancers harbor stem signatures that were previously found to, at least in part, be regulated by FOXO transcription factors [3, 4]. We hypothesized that FOXO‐driven gene expression programs may be essential to sustain BBC and GBM as is the case in ESCs and other stem cell contexts [6, 31, 32]. To investigate this hypothesis, the impact of inhibiting FOXO1 with AS1842856 (a small molecule inhibitor that binds/inactivates unphosphorylated FOXO1) was examined in BBC and GBM cells as a potential novel cancer chemotherapy. AS1842856 treatment‐induced apoptosis in numerous BBC and GBM cell lines.

## Methods

### Cell culture and drug treatments

Cell lines were obtained from ATCC (American Type Culture Collection, Manassas, VA) and grown under standard conditions [5% CO_2_, 10% FBS (fetal bovine serum)], with 5% antifungal/antibacterial (Anti/Anti, Thermo Fisher, Waltham, MA, USA). Cell lines were tested for Mycoplasma using the MycoAlert Mycoplasma Detection Kit (Lonza, Basel, Switzerland, cat: LT07‐218); all experiments were done with mycoplasma negative cells. U87MG cells were propagated in MEM (Minimal Essential Medium). BT549 and DBTRG cells were propagated in RPMI (Roswell Park Memorial Institute 1640 Medium). LN18, U118MG, A172, LN229, HCT116, and SW480 cells were propagated in DMEM (Dulbecco's Modified Eagle Medium). Neurosphere/cancer stem cell cultures for U87MG and BT549 cell lines were plated with 40 000 cells per mL in 3D Tumorsphere Medium XF (Sigma cat: C‐28070, Burlington, MA, USA). BT549 cancer stem cell cultures were supplemented with 1XB27 XenoFree CTS (Gibco/Thermo Fisher). AS1842856 was purchased from Calbiochem (Danvers, MA, USA) and utilized at 200 nm, 500 nm, and 1 μm final concentrations. AS1708727 was purchased from MedChemExpress (Monmouth Junction, NJ) and was used at 0.5, 1.0, and 2.0 μm concentrations. *5*‐*aza*‐2′‐deoxycytidine (5‐AZA) was purchased from Millipore/Sigma (Burlington) and utilized at a final concentration of 3 μm.

### RNAi experiments

MDA‐MB‐468 cells were grown to log phase in DMEM with 10% FBS without antibiotics. BT549 cells were grown to log phase in DMEM with 10% FBS without antibiotics. Cells were transfected with esiRNA (Sigma, St. Louis, MO, USA) *FOXO1* (EHU156591), *FOXO3* (EHU113611), *FOXO4* (EHU075731), or EGFP control esiRNA (*EHUEGFP*) using Lipofectamine 3000 (utilized only L3000 reagent, Invitrogen, Carlsbad, CA, USA). *FOXO1* RNAi or control was from Cell Signaling Technologies (cat: 6256 and 6568, respectively; Danvers, MA, USA) for BT549 cells in Fig. 4B; Sigma *FOXO1* esiRNA‐treated samples had the same gene expression results in BT549 cells (Fig. S3).

### Colony formation assays

Cells were plated at 2700 cells per mL and were treated for 5 days with the indicated drug. Treatments were investigated in triplicate (in numerous independent experiments) and stained with crystal violet. Plates were aspirated of media, then each well was washed with 1.0 mL of 1× phosphate‐buffered saline (PBS) once before being stained with 1.0 mL of crystal violet stain (0.5% crystal violet in buffered formalin) and incubated for 15 min. The stain was aspirated, and cells were washed three times with 0.5 mL of 1× PBS. After collections were completed, crystal violet‐stained plates were solubilized using 0.5 mL on each well of 10% acetic acid and placed on a shaker for 1 h. Solubilized samples were transferred to 96 well plates and quantified on a spectrophotometer at 590 nm using iMark Microplate Absorbance Reader (Bio‐Rad, Hercules, CA, USA). Quantified plates were analyzed with a Tukey Test. Error bars were added using the standard error.

### Western blot

Total protein was obtained from indicated cells by rinsing cells with 1× PBS (phosphate‐buffered saline) followed by directed lysis in 2× sample buffer (125 mm Tris–HCL at pH 6.8, 2% sodium dodecyl sulfate (SDS), 10% 2‐mercaptoethanol, 20% glycerol, 0.05% bromophenol blue, 8 m urea); 2× sample buffer was added to each well and cells scraped with a cell scraper. The lysate was collected from each well, placed into a 1.5 mL microcentrifuge tube, and heated for 10 min at 95 °C in a dry‐bath heat block. Protein lysates were separated by sodium dodecyl sulfate‐polyacrylamide gel electrophoresis (SDS/PAGE) at 100 V for 1 h. Resolved proteins were then transferred onto a polyvinylidene fluoride (PVDF) membrane for an hour and 30 min, then blocked in a 5% milk solution [Carnation powdered milk, 1× Tris‐buffered saline with Tween 20 (TBST)] for an hour. Membranes were incubated with indicated primary antibody overnight at 4 °C then washed for 20 min with TBST in 5‐min intervals. The blot was then incubated with a secondary antibody for 1.5 h. Membranes were washed for 20 min in 5‐min intervals and allowed to develop using SuperSignal West Dura Extended Duration Substrate luminol solution (Pierce Biotechnology, Waltham, MA, USA) for 5 min. A Bio‐Rad ChemDoc XRS+ Molecular Imager was utilized for protein detection (Bio‐Rad). Data were analyzed with NIH Image J. Antibodies were obtained from Cell Signaling Technologies: Cleaved Caspase 3 antibody (cat: 94530). Beta‐Actin antibody (clone AC‐74, cat: A2228) was obtained from Sigma and utilized at a 1 : 2000 dilution in TBST with 5% non‐fat dried milk.

### Quantitative Real‐Time PCR

Total RNA was prepared using the Qiagen RNeasy and DNAse kits (Hilden, Germany), then used to generate cDNA using Superscript Reverse Transcriptase II (Invitrogen). Samples (cDNAs) were analyzed using (Power SYBR Green Master Mix, Applied Biosystems, Foster City, CA, USA) and the ABI Step‐One Real‐time system (Carlsbad, CA, USA). Expression levels were normalized to *Beta‐Tub*ulin, *TUBB* (*Tubulin beta class I*) in gene expression experiments and calculated using $2^{-\Delta \Delta C_{T}}$ method [33]. Primer sequences are detailed in Table S1.

Work was performed with Institutional Biosafety Committee approval from the University of Texas‐Rio Grande Valley: Registration number: 2016‐003‐IBC.

## Results

### Inhibition of FOXO1 reduced BBC and GBM colony formation

**Fig. 1:**
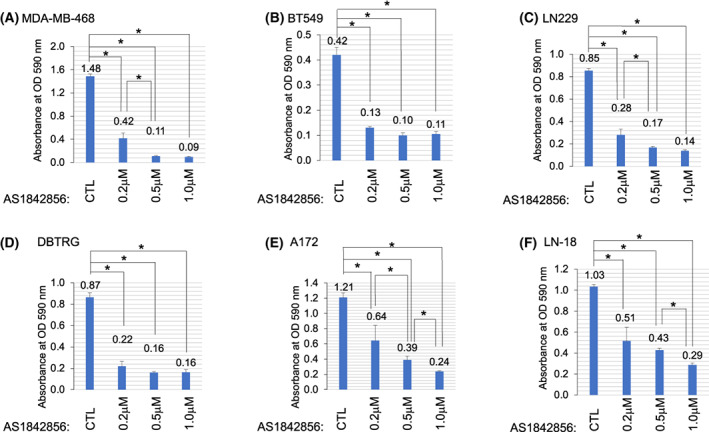
AS1842856 Treatment Reduced Colony Formation. (A–F) Indicated basal breast cancer (MDA‐MB‐468 or BT549) or GBM cell lines (LN229, DBTRG A172, or LN18) were treated with FOXO1 inhibitor AS1842856 for 5 days and subsequently stained with crystal violet. AS1842856 decreased colony formation in these cell lines. The results are representative of three independent experiments. Values in bar graphs are the mean with SEM. * denotes significantly different by the Tukey test compared with indicated samples (*P* < 0.05).

**Fig. 2:**
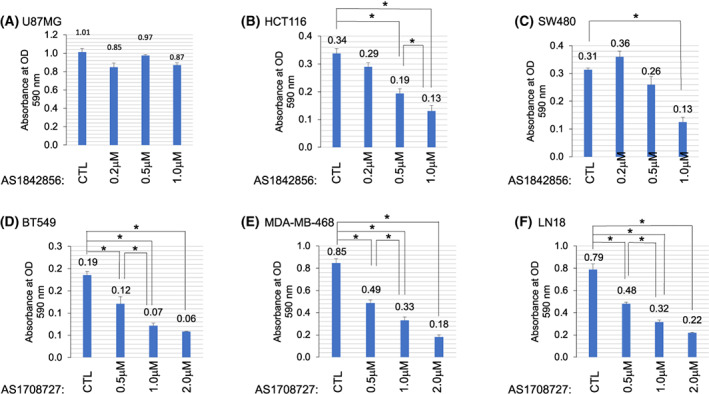
Inhibition of FOXO1 reduced colony number. (A) U87MG cells were treated with AS1842856; colony number was not significantly impacted. (B, C) Indicated colon cancer (HCT116 or SW480) cell lines were treated with FOXO1 inhibitor AS1842856 for 5 days and subsequently stained with crystal violet. AS1842856 decreased colony formation in these cell lines. (D–F) Treatment with another FOXO1 inhibitor AS1708727 in BT549, MDA‐MB‐468, and LN18 cells also led to reduced colony formation. The results are representative of three independent experiments. Values in bar graphs are the mean with SEM. * denotes significantly different by the Tukey test compared with indicated samples (*P* < 0.05).

AS1842856 is a selective FOXO1 inhibitor that reduces DNA binding and transactivation [34]. Given the role of FOXO1 in promoting stem gene expression in BBC and GBM cells, we examined the impact of FOXO1 inhibition on colony formation in a set of representative cancer cell lines. We treated BBC (MDA‐MB‐468 and BT549) and GBM (LN229, DBTRG, A172, LN‐18) cell lines with increasing drug 200 nm, 500 nm, and 1.0 μm for 5 days and stained cells with crystal violet. These cell lines were specifically chosen because our previous work showed that FOXO1 at least in part resided in the nuclei to drive stem gene expression in them [4]. Our hypothesis was that FOXO1 inhibition would be deleterious to these cells due to a reduction in stem gene expression. We found that AS1842856‐treated samples had fewer cells with 200 nm drug treatment with further reductions using 500 nm and 1 μm drug (Fig. 1A–F). U87MG growth was resistant to AS1842856 treatment (Fig. 2A). FOXO1 and ‐3 are required to maintain intestinal stem cells by preventing differentiation, suggesting a possible role in colon cancer [35]. To examine whether FOXO1 inhibition impacted colon cancer, we examined HCT116 and SW480 cell lines for colony formation upon AS1842856 treatment. We found significantly reduced colony formation with AS1842856 treatment in HCT116 and SW480 cell lines (Fig. 2B,C). Therefore, eight out of nine cell lines examined had reduced colony formation with FOXO1 inhibition using AS1842856. We also examined FOXO1 inhibitor, AS1708727 in several cell lines for the ability to impact colony formation [36]. AS1708727 treatment reduced colony formation in BT549, MDA‐MB‐468, and LN18 cell lines (Fig. 2D–F).

### Inhibition of FOXO1‐induced pro‐apoptotic genes in BBC and GBM cell lines

**Fig. 3:**
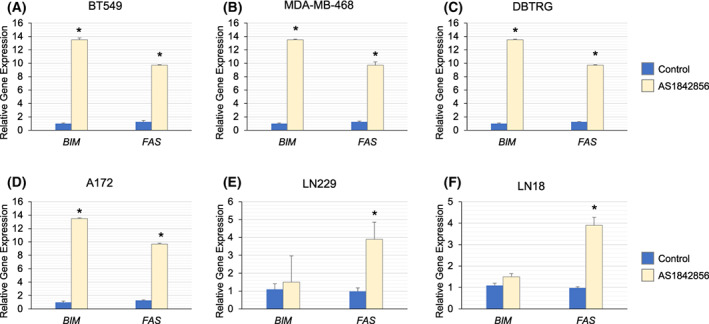
AS1842856 Treatment led to the induction of pro‐apoptotic genes. (A–F) Indicated cell lines were treated with 1 μm AS1842856 for 48 h and examined for changes in gene expression by qRT‐PCR using *TUBB* as the reference gene. We found that AS1842856 treatment‐induced apoptotic genes *FAS* and/or *BIM* in these cell lines. The results are representative of three independent experiments. Values in bar graphs are the mean with SEM. * denotes significantly different by the Tukey test compared with the control (*P* < 0.05).

To ascertain the mechanism responsible for reduced cell numbers upon FOXO1 inhibition, we performed qRT‐PCR analyses. We found that AS1842856 treatment for 48 h induced *FAS* and/or *BIM* gene expression in BT549, MDA‐MB‐468 breast cancer cell lines, and DBTRG, A172 LN229, LN18, and U87MG GBM cell lines (Fig. 3A–F and Fig. S1A). We also found that AS1842856 treatment for 48 h induced the *FAS* gene in HCT116 colon cancer cells (Fig. S1B).

Cancer stem cells (CSCs) are oftentimes resistant to chemotherapeutic interventions [37, 38, 39, 40]. To determine whether AS1842856 induced pro‐apoptotic genes in this setting, we isolated CSCs for BT549 and U87MG cells. We found that both that *FAS* and *BIM* were induced in CSCs upon AS1842856 treatment (Fig. S1C,D). CSCs were examined by qRT‐PCR for *FOXO1* gene expression. In comparison to control parent BT549 cells, derived CSCs cells had increased *FOXO1* gene expression (492‐fold) and an 8.2‐fold increase in the FOXO1 target gene *GADD45A* (Fig. S1E,F). In comparison to control parent U87MG cells, derived CSCs had less *FOXO1* gene expression (0.57‐fold) (Fig. S1E,F). U87MG CSCs had increased *GADD45A* expression (4.5‐fold). In both BT549 and U87MG cells, FOXO1 target gene *GADD45A* was increased in the CSCs compared with the control parent cell lines, suggesting that FOXO1 was more active in the CSCs (Fig. S1F). Gene expression of *FOXO1* was lower in U87MG CSCs (in comparison to parent U87MG cells) and was increased in BT549 CSCs (compared with parent cells). Commonly the expression level of FOXO transcription factors does not correlate with functional output, as these factors are heavily regulated by post‐transcriptional modifications such as phosphorylation and acetylation [22].

To further investigate the mechanism by which AS1842856 induced pro‐apoptotic genes, we performed western blot analyses for FOXO1 protein in treated BBC and GBM cell lines. We found that several cell lines (MDA‐MB‐468, LN‐18, and BT549) had increased FOXO1 protein after 48 h of 1 μm AS1842856 treatment, whereas other cell lines did not (DBTRG, A172, and LN229); see Fig. S2. The protein expression of FOXO1 did not consistently associate with increased *FAS* and *BIM* gene expression suggesting that other factors induce this increase.

To examine whether another FOXO1 inhibitor impacted pro‐apoptotic gene expression, we treated cells with AS1708727; a small molecular inhibitor identified in a high throughput screen to diminish FOXO1 transcriptional output measured with an IRE‐containing reporter gene [36]. Treatment of BT549 and MDA‐MB‐468 cells with AS1708727 for 4 days led to increased *FAS* gene expression (Fig. S1G,H).

**Fig. 4:**
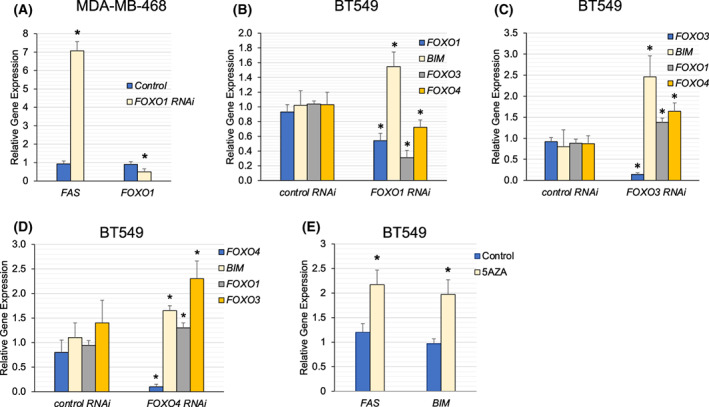
*FOXO* Factor RNAi Treatment led to the induction of apoptotic genes. (A, B) Indicated cell lines were treated with *FOXO1* RNAi or control (MDA‐MB‐468 72 h post‐transfection) or (BT549 18 h post‐transfection) and examined for changes in gene expression by qRT‐PCR using *TUBB* as the reference gene. We found that *FOXO1* RNAi treatment‐induced *FAS* in MDA‐MB‐468 cells. *FOXO1* RNAi samples had increased *BIM* and decreased *FOXO3* and *FOXO4* in BT549 cells. (C, D) *FOXO3* or *FOXO4* RNAi was examined in BT549 cells. (E) 5AZA treatment (3 μm for 48 h) led to the induction of *FAS*, and *BIM* in BT549 cells. The results are representative of three independent experiments. Values in bar graphs are the mean with SEM. * denotes significantly different by the Tukey test compared with the control (*P* < 0.05).

RNAi experiments were performed to assess whether the reduction of *FOXO1*‐induced apoptotic genes. We found that MDA‐MB‐468 cells treated with *FOXO1* esiRNA had increased *FAS* gene expression 72 h post‐transfection (Fig. 4A). This supports the notion that the reduction of *FOXO1* leads to the induction of apoptotic genes. RNAi in BT549 cells was also performed. Eighteen hours post *FOXO1*‐targeting RNAi transfection, *FOXO1* expression was decreased with increased *BIM expression in BT549 cells*; *FOXO3* and *FOXO4* were decreased in these *FOXO1* RNAi samples using two, independent RNAi kits (Fig. 4B and Fig. S3). The impact of *FOXO3* and *FOXO4* on *BIM* was also examined 18 h post‐RNAi transfection. We found that *FOXO3* RNAi samples had increased *BIM*, *FOXO1*, and *FOXO4* expression, whereas *FOXO4* RNAi samples had increased *BIM*, *FOXO1*, and *FOXO3* gene expression (Fig. 4C,D).

Given that *FAS* and *BIM* are commonly silenced in cancer to prevent apoptosis, we examined whether these genes were also silenced in basal breast cancer cells [41]. BT549 cells were treated with 5‐AZA to determine whether methylation impacted the expression of apoptotic genes in this context. We found that 5‐AZA treatment (3 μm for 48 h) induced *FAS* and *BIM* in BT549 cells (Fig. 4E). Perhaps FOXO1 promotes the silencing of apoptotic genes in these cancers.

### FOXO1 inhibition led to apoptosis induction based on caspase 3 cleavage and flow cytometric analyses

**Fig. 5:**
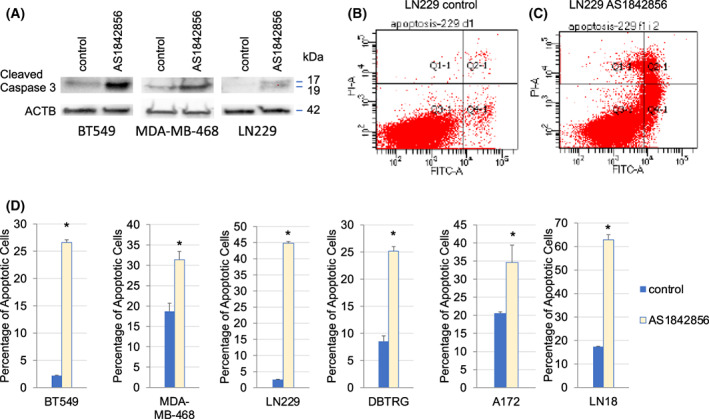
AS1842856 Treatment led to Apoptosis. (A) Western blot analyses were performed with BT549, LN229, and MDA‐MB‐468 samples that were treated with 1 μm AS1842856. These cell lines had increased caspase 3 cleavage. (B–D) Indicated cell lines were treated with 1 μm AS1842856, then stained with propidium iodide and FITC‐Annexin V followed by flow cytometric analyses. Apoptosis increased in LN229, BT549, MDA‐MB‐468, A172, DBTRG, and LN‐18 cells after AS1842856 treatment as measured by PI and/or Annexin V staining. The results are representative of three independent experiments. Values in bar graphs are the mean with SEM. * denotes significantly different by the Tukey test compared with the control (*P* < 0.05).

To clarify the impact of FOXO1 on BBC and GBM cell viability, we treated indicated cell lines with 1 μm AS1842856 and assessed Caspase 3 cleavage by western blot analysis. We found that AS1842856‐treated BT549, LN229, and MDA‐MB‐468 samples had increased caspase 3 cleavage (Fig. 5A); other cell lines (A172, DBTRG, and LN18) did not have detectable cleaved Caspase 3 (data not shown). To examine apoptosis using flow cytometry, we stained cells with Annexin V FITC to detect membrane‐exposed phosphatidyl‐serine indicating a loss in polarization and PI to detect cell permeability. We found that AS1842856‐treated cell lines including MDA‐MB‐468, BT549, LN229, DBTRG, A172, and LN‐18 had increased apoptosis (Fig. 5B–D) compared with vehicle control.

## Discussion

The role of FOXO1 in cancer and apoptosis is becoming increasingly complex [42]. Canonically FOXO transcription factors were described as tumor suppressors that induced apoptosis in part by increasing target gene expression, such as *TRAIL* [22, 23]. However, in DLBCL and AML (Acute Myeloid Leukemia), FOXO factors promote cancer aggressiveness in some instances by sustaining leukemic initiating cells [25, 43]. FOXO1, ‐3, and ‐4 are ubiquitously expressed and impact wide‐ranging biological processes, including metabolism, cell motility, cell fate, and the cell cycle [22, 23].

It remains unclear why inhibition of FOXO1 led to a loss in colony number accompanied by the induction of *FAS* in BBC and GBM cells. One possibility is a loss in cancer stem cells, leading to a loss in signals that prevent apoptosis. FOXO1 was required to sustain AML leukemic initiating cells [44]. Loss of FOXO1 led to differentiation and reduced cell numbers [43]. Another possibility (which is not mutually exclusive from the first possibility) is that FOXO1 is part of (or regulates) the machinery that silences *FAS* in BBC and GBM. Elegant experiments by Wajapeyee et al. delineated a step‐by‐step mechanism by which DNMT1 and other factors were recruited to the *FAS* promoter leading to cytosine methylation (among other things such as methylation of histone H3 on lysine 27) to silence this gene [41]. Indeed, *FAS* and *BIM* were induced by 5‐AZA treatment in BT549 cells, suggesting that methylation plays a role in its regulation in this setting (Fig. 4E). Perhaps like its ability to repress *CCND1*, FOXO1 directly represses *FAS* and *BIM* in BT549 cells [45, 46]. Alternatively, FOXO1 could regulate the expression of a required component of the machinery that silences *FAS*.

This work examined the impact of FOXO1 inhibition on BBC and GBM cells because this factor drives stem gene expression in these contexts [4, 5, 47]. We hypothesized that, like ESCs, FOXO1 would be required to sustain BBC and GBM cells [6]. Loss of function experiments indicates that at least in part, FOXO1 promotes the viability in a set of BBC and GBM cell lines (Fig. 1A–F). It is known that FOXO1 regulates stem genes, but the impact of these targets on cellular viability remains to be determined. Researchers have investigated the effects of cancer stem signaling on differentiated glioma cells using U87MG models that harbor oncogene *EGFR‐VIII* [48]. These cells secrete *LIF* and *IL6*, which are required to sustain cancer cell line growth and survival [48].

One confounding factor of this study was that while eight of the nine cell lines examined had reduced colony formation upon AS1842856 treatment, U87MG cells were resistant. Therefore, U87MG cells are resistant to AS1842856 treatment even though FOXO1 aids in driving stem genes in this cell line upon NVP‐BEZ235 (dual PI3K inhibitor) treatment. Notably, *FAS* was induced by AS1842856 treatment in U87MG cells (Fig. 2A). Hence, U87MG cells are resistant to apoptotic stimuli driven by a lack of FOXO1. Perhaps U87MG cells harbor a mutation that blocks apoptosis induction. U87MG cells are *TP53* wild type [49]. Alternatively, other FOXO factors such as FOXO3 may serve a functionally redundant role with FOXO1 to promote viability in U87MG cells. Ongoing efforts aim to examine biological roles for FOXO1 that underly BBC and GBM aggressiveness.

RNAi experiments were done to examine whether loss of *FOXO1*, ‐*3*, and ‐*4* could induce apoptotic genes. RNAi to each of these factors (individually) increased *BIM* gene expression in BT549 cells (Fig. 4B–D), highlighting functional overlap that is commonly observed with FOXO factors [22, 24]. Of note, *FOXO1* RNAi samples showed decreased *FOXO1*, ‐*3*, and ‐*4* gene expression at the 18‐h timepoint, suggesting that either there were off‐target effects or *FOXO1* promoted the expression of *FOXO3* and *FOXO4*. We examined an additional *FOXO1* RNAi sequence and found the same reductions in *FOXO*3, and ‐*4* with increased *BIM* (Fig. S3), hinting that this was not an off‐target effect. One possibility is that FOXO1 drives a positive feedback loop to sustain *FOXO3* and *FOXO4*. In contrast to FOXO1 reduction, *FOXO3* or *FOXO4* RNAi led to increases in the other examined FOXO factors. For example, *FOXO3* RNAi samples had increased *FOXO1* and *FOXO4* expression (Fig. 4C). Taken together, these data highlight functional interactions between the FOXO factors in BT549 cells and a possible distinct role for FOXO1 as a driver of *FOXO3* and ‐*4* gene expression in this setting.

We noted that AS1708727 only induced the *FAS* gene after 4 days of treatment, whereas AS1842856 robustly caused *FAS* and *BIM* in the same cell lines BT549 and MDA‐MB‐468 (Fig. 3A–F and Fig. S1G,H). Other researchers have published more robust impacts on gluconeogenesis genes with AS1842856 than AS1708272; albeit this conclusion compares data from separate studies in distinct contexts [34, 36].

This work is the first to highlight FOXO1 as a possible therapeutic target in the poor‐prognosis cancers BBC and GBM. Inhibition of FOXO1 by AS1842856 or AS1708727 treatment led to reduced colony formation and apoptotic gene expression. These data reveal novel avenues for therapeutics and insights into the functions of FOXO1 in these cancers.

## Conflict of interest

U.S. Provisional Patent Application No. 63271289 Entitled: ‘FOXO1‐Targeted Therapy For The Treatment Of Cancer’ by Megan Keniry et al. was filed on 10/25/2021.

## Author contributions

DF, AL, MK, and SU performed experiments. DF, AL, SU, MK, and BG developed concepts and critically analyzed data. DF and MK wrote the manuscript.

## Supporting information


**Fig. S1.** AS1842856 treatment led to the induction of pro‐apoptotic genes. (A‐D) Indicated cell lines were treated with 1 μM AS1842856 for 48 h and examined for changes in gene expression by qRT‐PCR using *TUBB* as the reference gene. We found that AS1842856 treatment‐induced apoptotic genes *FAS* and/or *BIM* in U87MG, and HCT116 cell lines as well as in BT549 cancer stem cells (CSCs) and U87MG CSCs. (E) *FOXO1* gene expression was assessed by qRT‐PCR. (F) *GADD45A* gene expression was assessed by qRT‐PCR. (G‐H) (C‐D) Treatment with AS1708727 led to *FAS* induction in BT549 (1 μM treatment for 4 days) and MDA‐MB‐468 cells (1 μM treatment for 2 days). The results are representative of three independent experiments. Values in bar graphs are the mean with SEM. * denotes significantly different by the Tukey test compared with control (*P* < 0.05).

**Fig. S2.** AS1842856 treatment had varied impacts on FOXO1 protein expression in BBC and GBM cell lines. Indicated cell lines were treated with 1 μM AS1842856 for 48 h and analyzed by western blot analysis.

**Fig. S3.** *FOXO1* RNAi treatment led to the induction of *BIM*. *FOXO1* RNAi samples had increased *BIM* and decreased *FOXO3* and *FOXO4* in BT549 cells. These samples were treated with *FOXO1* esiRNA and samples collected 18 h post‐transfection. The results are representative of three independent experiments. Values in bar graphs are the mean with SEM. * denotes significantly different by the Tukey test compared with control (*P* < 0.05).

**Table S1.** Gene‐specific PCR primers.

## Data Availability

All cell lines and additional data prepared from this work are available upon request.
