# Supplementary material for: The FOXO1 inhibitor AS1842856 triggers apoptosis in glioblastoma multiforme and basal‐like breast cancer cells
Source: FEBS Open Bio. 2023 Jan 16;13(2):352–62. doi: 10.1002/2211-5463.13547 (PMC9900086; doi:10.1002/2211-5463.13547)
Supplement: Supplementary file 1 — Fig. S1. AS1842856 treatment led to the induction of pro‐apoptotic genes. (A‐D) Indicated cell lines were treated with 1 μM AS1842856 for 48 h and examined for changes in gene expression by qRT‐PCR using TUBB as the reference gene. We found that AS1842856 treatment‐induced apoptotic genes FAS and/or BIM in U87MG, and HCT116 cell lines as well as in BT549 cancer stem cells (CSCs) and U87MG CSCs. (E) FOXO1 gene expression was assessed by qRT‐PCR. (F) GADD45A gene expression was assessed by qRT‐PCR. (G‐H) (C‐D) Treatment with AS1708727 led to FAS induction in BT549 (1 μM treatment for 4 days) and MDA‐MB‐468 cells (1 μM treatment for 2 days). The results are representative of three independent experiments. Values in bar graphs are the mean with SEM. * denotes significantly different by the Tukey test compared with control (P < 0.05). [file FEB4-13-352-s004.pdf]

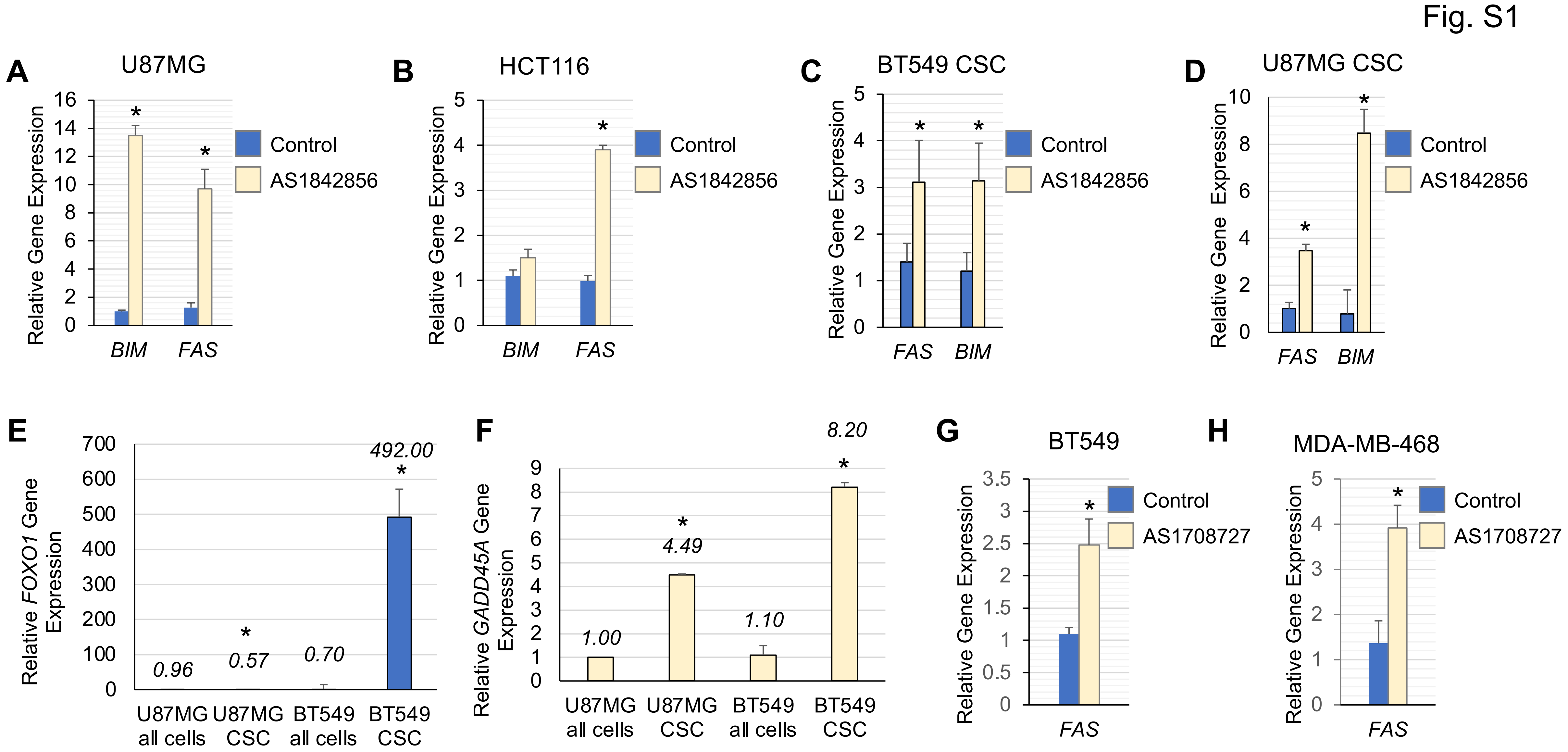

Fig. S1. AS1842856 Treatment led to the induction of pro-apoptotic genes. (A-D) Indicated cell lines were treated with 1μM AS1842856 for 48 hours and examined for changes in gene expression by qRT-PCR using *TUBB* as the reference gene. We found that AS1842856 treatment induced apoptotic genes *FAS* and/or *BIM* in U87MG and HCT116 cell lines as well as in BT549 cancer stem cells (CSCs) and U87MG CSCs. (E) *FOXO1* gene expression was assessed by qRT-PCR. (F) *GADD45A* gene expression was assessed by qRT-PCR. (G-H) Treatment with AS1708727 led to *FAS* induction in BT549 (1μM treatment for 4 days) and MDA-MB-468 cells (1μM treatment for two days). * denotes significantly different by Tukey Test compared to control ($P<0.05$).
