# Supplementary material for: The FOXO1 inhibitor AS1842856 triggers apoptosis in glioblastoma multiforme and basal‐like breast cancer cells
Source: FEBS Open Bio. 2023 Jan 16;13(2):352–62. doi: 10.1002/2211-5463.13547 (PMC9900086; doi:10.1002/2211-5463.13547)
Supplement: Supplementary file 2 — Fig. S2. AS1842856 treatment had varied impacts on FOXO1 protein expression in BBC and GBM cell lines. Indicated cell lines were treated with 1 μM AS1842856 for 48 h and analyzed by western blot analysis. [file FEB4-13-352-s002.pdf]

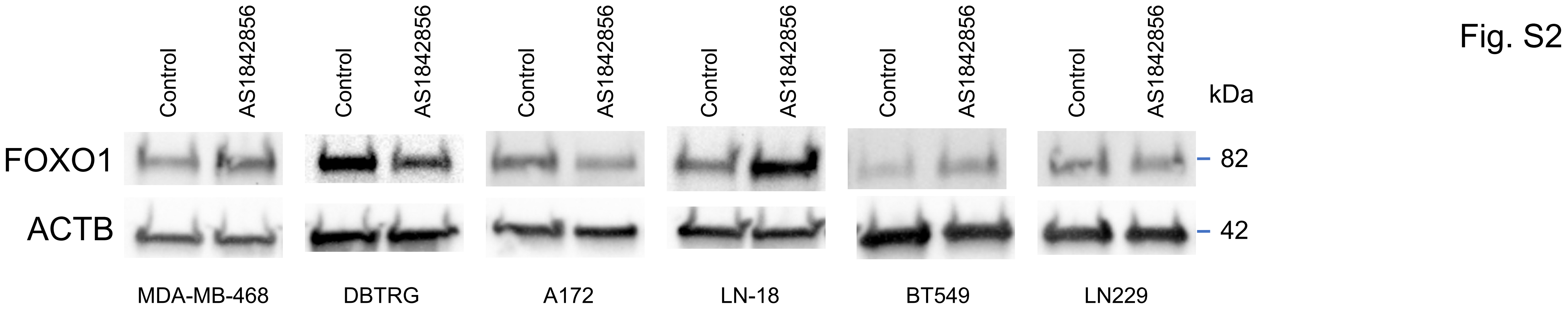

Fig. S2 AS1842856 treatment had varied impacts on FOXO1 protein expression in BBC and GBM cell lines. Indicated cell lines were treated with 1 micromolar AS1842856 for 48 hours and analyzed by western blot analysis. The actin control for MDA-MB-469 is the same as in Figure 5A.
