# Supplementary material for: The FOXO1 inhibitor AS1842856 triggers apoptosis in glioblastoma multiforme and basal‐like breast cancer cells
Source: FEBS Open Bio. 2023 Jan 16;13(2):352–62. doi: 10.1002/2211-5463.13547 (PMC9900086; doi:10.1002/2211-5463.13547)
Supplement: Supplementary file 3 — Fig. S3. FOXO1 RNAi treatment led to the induction of BIM. FOXO1 RNAi samples had increased BIM and decreased FOXO3 and FOXO4 in BT549 cells. These samples were treated with FOXO1 esiRNA and samples collected 18 h post‐transfection. The results are representative of three independent experiments. Values in bar graphs are the mean with SEM. * denotes significantly different by the Tukey test compared with control (P < 0.05). [file FEB4-13-352-s003.pdf]

Fig. S3

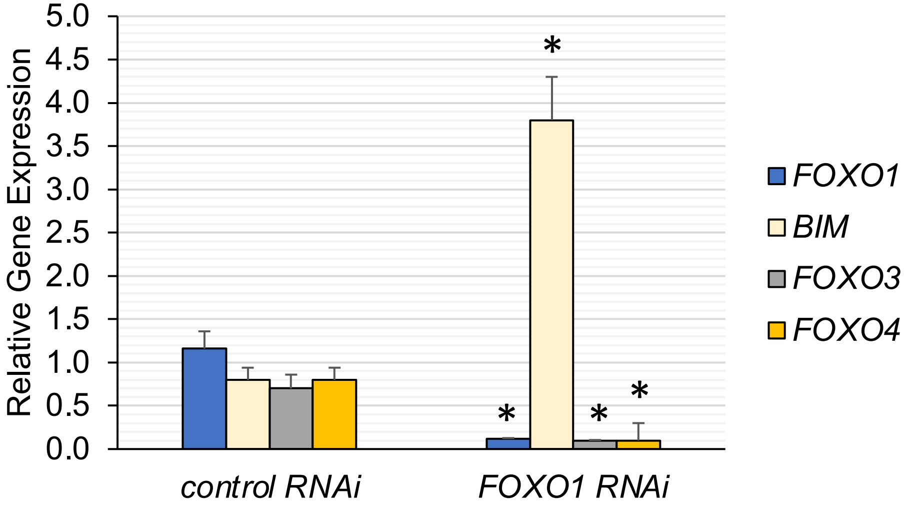

Fig. S3 *FOXO1* RNAi Treatment led to the induction of *BIM*. *FOXO1* RNAi samples had increased *BIM* and decreased *FOXO3* and *FOXO4* in BT549 cells. These samples were treated with *FOXO1* esiRNA and samples collected 18-hours post-transfection. * denotes significantly different by Tukey Test compared to control (*P*<0.05).
