# Supplementary material for: The FOXO1 inhibitor AS1842856 triggers apoptosis in glioblastoma multiforme and basal‐like breast cancer cells
Source: FEBS Open Bio. 2023 Jan 16;13(2):352–62. doi: 10.1002/2211-5463.13547 (PMC9900086; doi:10.1002/2211-5463.13547)
Supplement: Supplementary file 4 — Table S1. Gene‐specific PCR primers. [file FEB4-13-352-s001.docx]

**Supplemental Table S1. Gene-specific PCR primers**

The primers used for gene expression analysis are detailed.

| Primer | Sequence |
| --- | --- |
| *FAS* F (detects *FAS* cell surface death receptor) | 5’ GGGGTGGCTTTGTCTTCTTCTTTTG 3’ |
| *FAS* R (detects *FAS* cell surface death receptor) | 5’ ACCTTGGTTTTCCTTTCTGTGCTTTCT’ 3 |
| *BIM* F (*BCL2L11*) | 5’ CAAACCCCAAGTCCTCCTT 3’ |
| *BIM* R (*BCL2L11*) | 5’ TCTTGGGCGATCCATATCTC 3’ |
| *TUBB* F | 5’ CTGGACCGCATCTCTGTGTA 3’ |
| *TUBB* R | 5’ ATCTGGCCAAAAGGACCTG 3’ |
| *FOXO1* F | 5’ ACGAGTGGATGGTCAAGAGC 3’ |
| *FOXO1* R | 5’ CTGCACACGAATGAACTTGC 3’ |
| *FOXO3* F | 5’ CTTCAAGGATAAGGGCGACA 3’ |
| *FOXO3* R | 5’ TCTTGCCAGTTCCCTCATTC 3’ |
| *FOXO4* F | 5’ AGGACAAGGGTGACAGCAAC 3’ |
| *FOXO4* R | 5’ GGTGGCCTCGTTGTGAAC 3’ |
